# Diagnostic efficacy of cystatin-c in association with different ACE genes predicting renal insufficiency in T2DM

**DOI:** 10.1038/s41598-023-32012-w

**Published:** 2023-03-31

**Authors:** Mona Mohamed Taha, Heba Mahdy-Abdallah, Eman Mohamed Shahy, Mona Adel Helmy, Lamia Samir ElLaithy

**Affiliations:** Department of Environmental and Occupational Medicine, National Research Centre, Dokki, Cairo, Egypt

**Keywords:** Biochemistry, Nephrology

## Abstract

Diabetic nephropathy (DN) seems to be the major cause of chronic kidney disease that may finally lead to End Stage Renal Disease. So, renal function assessment in type 2 diabetes mellitus (T2DM) individuals is very important. Clearly, DN pathogenesis is multifactorial and different proteins, genes and environmental factors can contribute to the onset of the disease. We assessed sensitive and specific biomarkers (in blood and urine) which can predict kidney disease susceptibility among T2DM patients. Serum cystatin-c (cyst-c) in blood and urinary hemeoxygenase (HO-1) in addition to ACE I/D polymorphism and ACE G2350A genotypes. Hundred and eight T2DM patients and 85 controls were enrolled. Serum cystatin-c and urinary (HO-1) were tested by ELISA. Genetic determination of both ACE I/D polymorphism and ACE G2350A genotypes was performed by PCR for all participants. Significant rise in serum cystatin-c and urinary HO-1 levels were shown in diabetic groups compared with control group. Moreover, GG genotype of ACE G2350A gene in diabetic group was associated with rise in serum cystatin-c and urinary HO-1 compared with control group. Mutant AA genotype demonstrated increase in urinary HO-1. DD polymorphism was associated with rise in serum creatinine and cyst-c in diabetic group. Positive correlation was seen between duration of diabetes and serum cyst-c and between serum glucose and urinary (HO-1) in diabetic group. The results from this study indicated an association of serum cystatin-c with GG genotype of ACE G2350A in conjugation with DD polymorphism of ACE I/D which could be an early predictor of tubular injury in T2DM diabetic patients.

## Introduction

Diabetes mellitus (DM) represents one of the most common chronic metabolic diseases with increasing prevalence^1^. In T2 DM, diabetic nephropathy (DN), where proteinuria is the main characteristic, represents one of the most frequent microvascular complication^2^. As early intervention, diagnostic markers could detect DN at early stage. It can slow kidney function deterioration and aid in reducing adverse outcomes. Pathologically, kidney hypertrophy is usually shown in DN patients with basement membrane thickening, extracellular matrix proteins deposition, glomerular sclerosis and interstitial fibrosis^2,3^. Although follow up of DN patients can be done by blood glucose and blood pressure control, progression of renal failure can eventually be seen in many patients^4^. Hence, understanding DN pathophysiology and development of new biomarkers seem to be of high demand for DN early diagnosis.

Cystatin-c, (a low molecular weight protein 13,343 Da), is an endogenous cysteine protease inhibitor that is produced in most tissues. It is found also in all biological fluids^5^. It is synthesized by all nucleated cells at a stable rate and different factors such as sex, protein ingestion, inflammation, or muscle mass had no impact on its concentration^6^. It represents an ideal marker of endogenous glomerular filtration rate. For assessment of renal function, cystatin-c represents a more sensitive parameter than serum creatinine^5^. In critically ill adults, it is associated with recovery prognosis of acute kidney injury^5–7^. In addition, Taha et al.^8^ deduced that, in cadmium nephrotoxicity, cystatin-c is the most applicable biomarker.

Heme oxygenase 1 (HO-1) is known as an inducible enzyme which has different properties e.g., potent antioxidant, anti-inflammatory and anti-apoptotic one. Its activity was shown to occur via breakdown of heme fraction and generate protective products like carbon monoxide (CO) and biliverdin, with subsequent formation of bilirubin and ferritin via iron release from the heme part^9,10^. Under homeostatic conditions, low levels of HO-1 protein in found in the tubules. While under several stress conditions that include oxidative stress, heat shock, hypoxia, heavy metals and toxins, heterogenous expression of HO-1 is detected in different renal compartments. In the medulla HO-1 is more highly expressed than the cortex, while in tubules, its expression is found to be very strong (especially proximal tubules). In contrary to glomeruli where minimal or absence in HO-1 expression^11^. At present, in DN, assessment of urinary heme oxygenase-1 (uHO-1) was close to tubular damage^12^. Upregulation of HO-1 in proximal tubule cells seems to be an efficient biomarker of intrarenal activity as well as renal injury^13^. Recent study showed that, in patients with various kidney diseases, HO-1 levels within damaged cells falls away into the renal tubules^14^.

Angiotensin I-converting enzyme (ACE) is a key component of renin–angiotensin system (RAS). Its synthesis was observed in both epithelial and endothelial cells that is located in different organs as lungs, kidneys and blood vessels. Its main function is to catalyze producing vasoactive peptide angiotensin II from angiotensin I (its precursor), in addition to inactivate bradykinin. Besides, angiotensin II has a critical role in maintaining blood pressure, sodium homeostasis and renal hemodynamics. Mahwish et al.^15^ reported that ACE gene is located on chromosome 17q23 and spans approximately 21 kb. This gene was shown to include 26 exons as well as 25 introns and encodes a protein containing 1,306 amino acid. Previous records of National Center for Biotechnology Information (NCBI) had shown that ACE gene had more than 160 polymorphisms (mainly single nucleotide polymorphisms (SNP)^16^. Within the circulation, ACE G2350A gene (one of the ACE genes) had demonstrated a significant role in the determination of ACE level^8^.

Other polymorphism is the insertion/deletion (I/D) one which represents the most widely studied ACE polymorphism. It is characterized by the presence of a 287 bp Alu repeat sequence (insertion) or absence (deletion) in intron^16^. This produces three genotypes (wild I/I homozygous, heterozygous I/D heterozygote, and mutant homozygous D/D)^17,18^. It was recorded in individuals with DD genotype, higher concentration of ACE in both tissue and plasma ACE in comparison with either ID of II genotypes^19,20^.

Silveira et al.^18^ revealed that ACE insertion (I)/deletion (D) polymorphism could cause instability of RAS as well as dysregulation of micro and macrocirculation and electrolytic homeostasis of the kidney. Moreover, it could affect or lead to DN through a direct effect on cellular hypertrophy resulting vascular modulation in the kidney.

Our goal is to investigate and assess serum cystatin-C and urinary HO-1 in the early diagnosis of renal injury (as biomarkers of renal dysfunction) and its association with ACE I/D and G2350A genotypes in T2DM patients.

## Results

Table 1 mentioned to gender distribution, age, smoking habit as well as duration of smoking in both groups (diabetic patients and control groups). Duration of diabetes was shown to be less than 15 years.

**Table 1:** Demographic data of participants in diabetic patients and control groups.

	Diabetic group N = 108	Control group N = 85
Gender		
Male	60 (55.6%)	63 (74.1%)
Female	48 (44.4%)	22 (25.9%)
Age (years)	54.1 ± 9.44	52.14 ± 7.61
Duration of diabetes (years)	15.32 ± 5.83	–
Smoking habit		
Non smoker	85 (78.7%)	56 (65.9%)
Ex-smoker	8 (7.4%)	2 (2.4%)
Smoker	15 (13.9% )	27 (31.7%)
Smoking duration (years)	20.28 ± 11.82	17.19 ± 9.9

Table 2 illustrated high significant rise in serum glucose levels, cystatin-c and urinary hemeoxygenase, as well as significant decline in eGFR in diabetic groups compared with control group. Serum creatinine levels recorded non-significant change.

**Table 2:** Levels of biochemical parameters in diabetic and control groups.

	Diabetic group N = 108	Control group N = 85	t test	*p* value
Serum glucose (mg/dL)	164.43 ± 79.69	88.86 ± 18.25	8.6	0.001**
HbA_1_C (%)	7.31 ± 5.40	4.64 ± 0.71	4.5	0.001**
Serum creat (mg/dL)	1.14 ± 0.66	0.83 ± 0.12	1.84	0.07
Serum Cyst-C (µg/L)	779.67 ± 636.93	520.24 ± 197.97	3.6	0.001**
Urinary HO-1 (ng/mg creat)	16.36 ± 11.45	12.46 ± 12.0	2.24	0.026*
eGFR (mL/min/1.73m^2^)	105.97 ± 37.92	122.74 ± 28.39	3.8	0.001*

**High significant *p* < 0.001, *significant at *p* < 0.05.

Table 3 revealed high significant difference in distribution in genotype frequency of ACE G2350A gene (*p* < 0.003) and significant distribution in ACE I/D gene (*p* < 0.049) in diabetic patient compared with control group.

**Table 3:** Genotypes and allelic frequencies of ACE I/D and ACE G2350A genes in diabetic Patient and control groups.

Genotypes/alleles	Diabetic patients group (n = 108)	Control group (n = 85)	χ^2^	*P* value
ACE I/D				
Genotype (n)				
II	11 (10.2%)	7 (8.2%)	6.03	0.049*
ID	60 (55.6%)	34 (40%)		
DD	37 (34.2%)	44 (51.8%)		
Allele (2n)				
D	134	122		
I	82	48		
ACE (G2350A)				
Genotype (n)				
GG	33 (30.6%)	47 (55.3%)	14.8	0.001**
GA	47 (43.5%)	30 (35.3%)		
AA	28 (25.9%)	8 (9.4%)		
Allele (2n)				
G	113 (52.3%)	124 (72.9%)		
A	103 (47.7%)	46 (27.1%)		

**High significant *p* < 0.001, *significant at *p* < 0.05.

Table 4 found significant difference in levels of HbA_1_C (%) and serum glucose in all genotypes of ACE G2350A gene. Also, GG genotype of ACE G2350A gene in diabetic patient group was associated with significant increase in levels of both serum cystatin-c and urinary HO-1. Mutant genotype AA was associated with significant increase in urinary HO-1 levels, while heterozygote genotype GA was associated with significant fall in eGFR levels in diabetic subjects compared with control ones.

**Table 4:** Levels of studied biochemical parameters in different ACE2 G2350A genotypes in diabetic patient and control groups.

	GG		GA		AA	
	Diabetic patient (n = 32)	Control (n = 43)	Diabetic patient (n = 44)	Control (n = 29)	Diabetic patient (n = 26)	Control (n = 8)
Serum glucose (mg/dL)	174.44 ± 76.10	84.54 ± 17.28	160.80 ± 87.0	92.87 ± 16.71	140.88 ± 57.43	97.13 ± 20.86
*p* value	0.001**		0.001**		0.048*	
HbA_1_C (%)	7.05 ± 1.02	4.49 ± 0.67	8.2 ± 7.90	4.72 ± 0.64	6.72 ± 1.33	5.39 ± 0.56
*p* value	0.001**		0.001**		0.009**	
Serum creatinine (mg/dL)	0.88 ± 0.20	0.83 ± 0.12	1.05 ± 0.65	0.83 ± .016	1.11 ± 1.0	0.8 ± 0.09
*p* value	0.13		0.18		0.37	
Serum cyst-c (µg/L)	703.88 ± 382.24	527.28 ± 217.83	787.45 ± 635.04	525.90 ± 187.37	737.54 ± 729.84	521.63 ± 163.10
*p* value	0.014*		0.069		0.47	
Urinary HO-1 (ng/mg creat)	16.35 ± 9.63	10.66 ± 10.20	14.36 ± 12.58	13.17 ± 7.41	21.91 ± 11.63	13.0 ± 12.0
*p* value	0.016*		0.57		0.017*	
eGFR(mL/min/1.73m^2^)	111.5 ± 28.78	121.14 ± 24.06	102.28 ± 37.81	125.96 ± 35.99	105.85 ± 46.21	117.0 ± 26.42
*p* value	0.16		0.014*		0.4	

**High significant *p* < 0.001, *significant at *p* < 0.05.

Table 5 found significant difference in levels of HbA_1_C (%) and serum glucose in all ACE I/D polymorphism. Also, DD polymorphism was associated with significant increase in levels of serum creatinine and cyst-c in diabetic patient group. Moreover, I/D polymorphism was associated with significant increase in urinary HO-1 levels and high significant decline in eGFR levels in diabetic patients compared with control group.

**Table 5:** Levels of studied biochemical parameters in different ACE I/D genotypes in diabetic patient and control groups.

	DD		ID		II	
	Diabetic patient (n = 35)	Control (n = 41)	Diabetic patient (n = 57)	Control (n = 32)	Diabetic patient (n = 10)	Control (n = 7)
Serum glucose (mg/dL)	167.77 ± 84.05	89.73 ± 17.64	153.71 ± 66.01	87.78 ± 19.77	171.20 ± 113.28	88.14 ± 9.80
*p* value	0.001**		0.001**		0.046*	
HbA_1_C (%)	7.03 ± 1.91	4.62 ± 0.78	7.63 ± 7.03	4.64 ± 0.63	6.73 ± 2.23	4.9 ± 0.58
*p* value	0.001**		0.003**		0.05*	
Serum creatinine (mg/dL)	1.07 ± 0.72	0.81 ± 0.11	1.02 ± 0.70	0.85 ± 0.15	0.8 ± 0.19	0.78 ± 0.16
*p* value	0.027*		0.09		0.79	
Serum cyst-c (µg/L)	674.29 ± 395.50	497.22 ± 162.21	812.86 ± 716.84	568.84 ± 247.22	641.50 ± 275.73	492.86 ± 138.53
*p* value	0.011*		0.06		0.17	
Urinary HO-1 (ng/mg creat)	15.477 ± 13.50	15.35 ± 8.62	16.82 ± 10.85	11.64 ± 10.43	13.28 ± 5.66	9.53 ± 6.51
*p* value	0.96		0.033*		0.23	
eGFR(mL/min/1.73m^2^)	110.91 ± 41.78	122.77 ± 25.53	99.0 ± 34.12	123.0 ± 33.69	125.36 ± 37.88	121.33 ± 21.38
*p* value	0.14		0.003**		0.78	

**High significant *p* < 0.001, *significant at *p* < 0.05.

**Figure 1:**
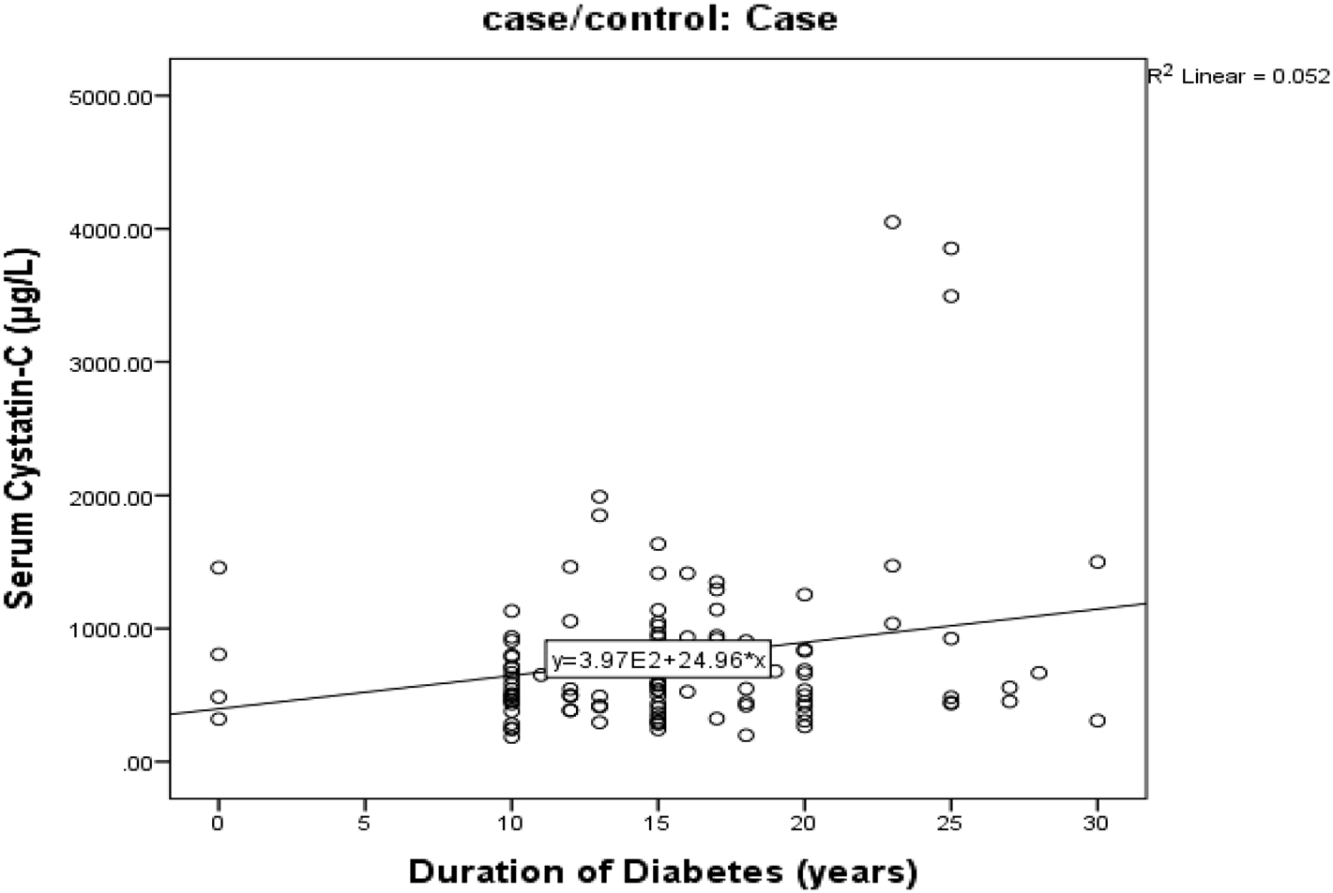
Correlation between duration of diabetes and level of serum cystatin-c in diabetic patient group.

Figure 1 showed significant positive correlation between duration of diabetes and level of serum cystatin-c in diabetic patient group (r = 0.22, *p* < 0.027).

**Figure 2:**
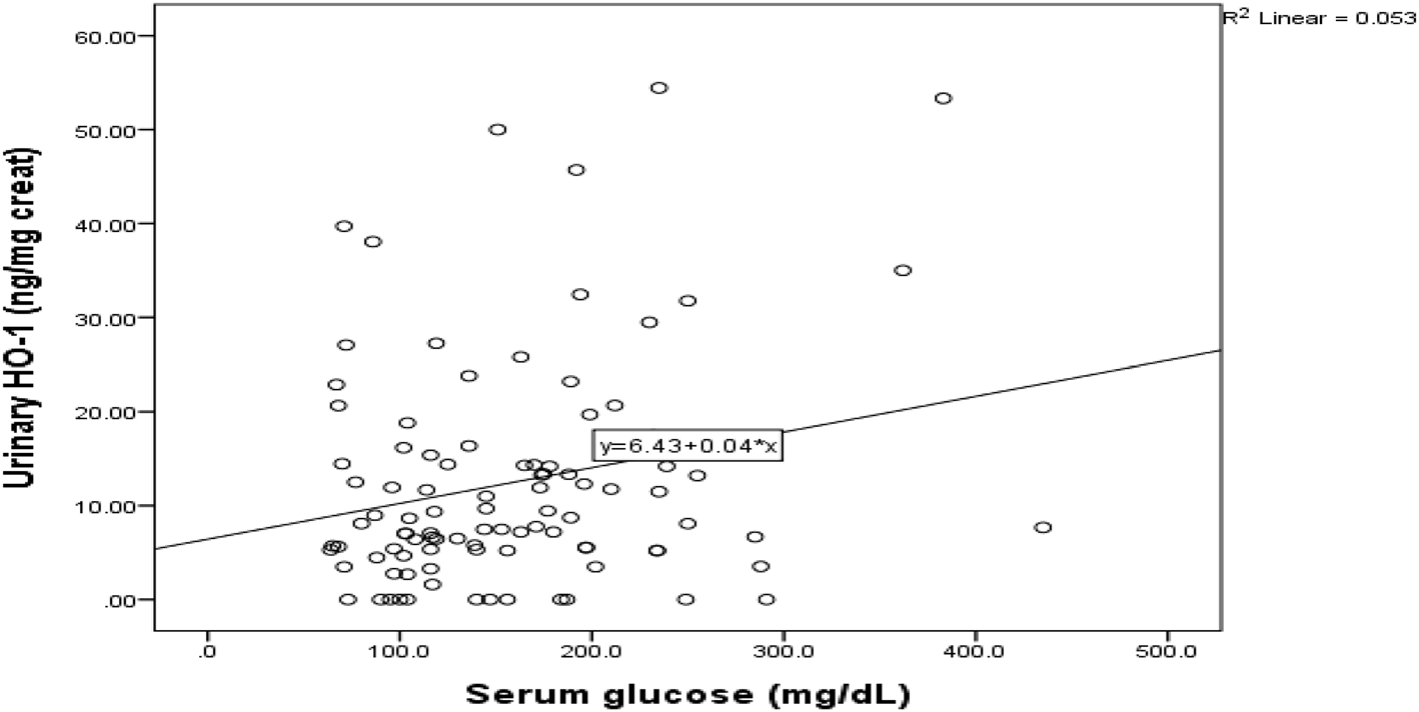
Correlation between level of urinary hemeoxygenase and serum glucose in diabetic patient group.

Figure 2 illustrated a significant positive correlation between level of urinary hemeoxygenase and serum glucose in diabetic patient group (r = 0.24, *p* < 0.02).

**Figure 3:**
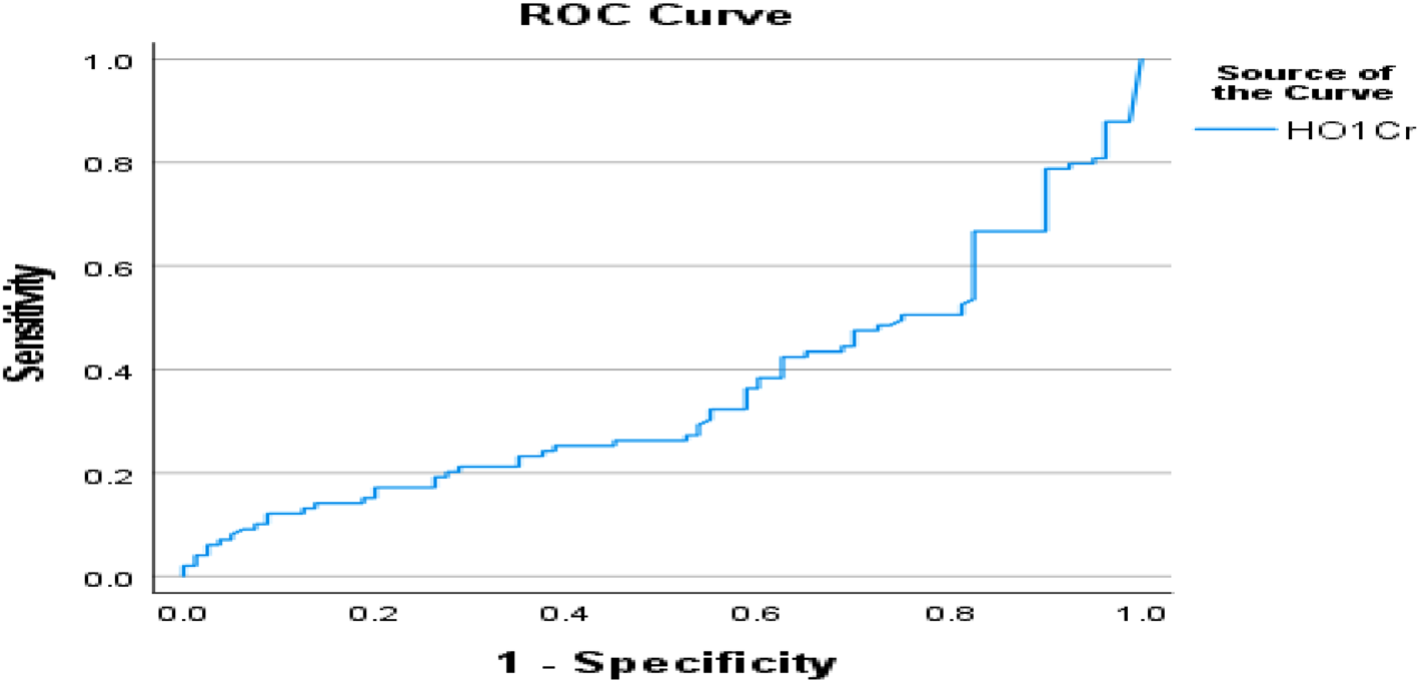
The crude AUC of the ROC curve of urinary HO-1 for predicting early tubular injury in diabetic patients.

Figure 3 mentioned that crude area under the ROC curve (AUC) of urinary hemeoxygenase was 0.356, (*p* < 0.001) to predict early tubular injury in diabetic kidney, suggesting that urinary HO-1 could represent as potent biomarker of DN in urine.

**Figure 4:**
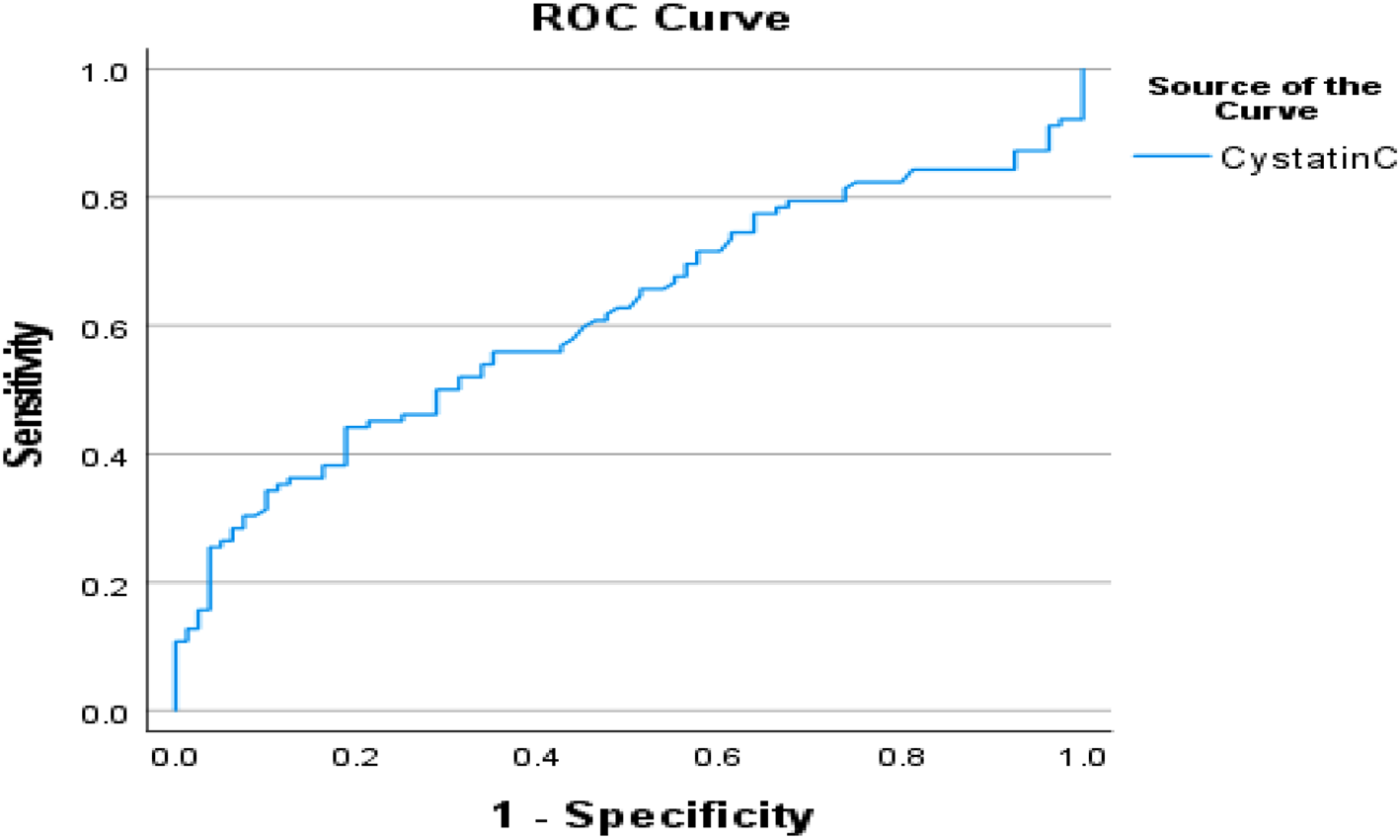
The crude AUC of the ROC curve of serum cystatin-c for predicting early tubular injury in diabetic patients.

Figure 4 showed that crude area under the ROC curve (AUC) of serum cystatin-c was 0.617, (*p* < 0.005) for predicting early tubular injury in diabetic kidney suggesting that serum cystatin-c could be a potential biomarker of DN.

## Discussion

Diabetic kidney disease (DKD) accounted for the increased risk of mortality among diabetic patients and eventually was recorded in about half of diabetic patients with type 2 diabetes mellitus (T2DM)^21^. Our study had focused on many markers such as serum cystatin-C and urinary hemeoxygenase to be screened as early indicators of DN in long-term type 2 diabetic patients.

Significant rise in serum cystatin-c levels with significant decline in eGFR in diabetic group was recorded in current study in comparison with control group. Our results were in accordance with Saptoka et al.^6^. Also, other study confirmed that cystatin-c is a well-defined marker which is advised to be assessed for early DN diagnosis and progression^6,22^. As serum creatinine was unable to detect early decline in GFR owing to the fact that levels of serum creatinine begins to rise above normal range only in case of kidney had lost approximately 50% of its function. This proposes that serum cystatin-c levels are related to impairment in tubular function and in turn can be suggested as an early marker of kidney diseases^23^. Moreover, detecting CKD at early stage could be important for early intervention in order to be able to slow kidney function loss, which in turn helps improving survival and quality of life.

Regarding urinary HO-1, our results were in line with Abd ElHameed and Saied^14^ who referred that hyperglycemia and diabetic substrates in the form of glycation end products can affect renal tubular cells. That results tubular cell hypertrophy and interstitial deposition of chemokines, cytokines and adhesion molecules leading to tubular inflammation and fibrosis^24^. HO-1 protein was upregulated in renal tubules but not in glomerular cells in affected (diseased) kidney. This upregulation may be due to the difference in both sensitivity and response to oxidant stress exhibited by these cells^14^.

Our results demonstrated significant difference in genotypic and allelic frequencies distributions of ACE I/D and G2350A polymorphisms between diabetic and control groups with dominant heterozygote polymorphisms were shown in both types of genes (I/D and GA) in diabetic patient group Table 3. Also, in ACE I/D polymorphism, the carriers of the I allele and A allele of ACE G2350A were more frequent in the diabetic patients than the control group. Furthermore, Verma et al.^25^ reported that patients with AA genotype were at lower risk for CKD in comparison with those with GG genotype. In current study, A-allele exhibited lower significant frequency in diabetic cases.

Present study recorded an association of GG genotype of ACE G2350A gene in diabetic patient group with rise in both serum cystatin-c and urinary heme oxygenase levels. Another study showed significant association between the G allele for the ACE G2350A gene polymorphism with higher blood pressure as well as ACE concentration. This association could induce nephropathy^26^. While mutant AA genotype was associated with increase in urinary HO-1 that may confirm, as Narita et al.^27^ stated that higher risk for progressive renal dysfunction may be found in patients with AA genotype of the G2350A polymorphism. Furthermore, heterozygote genotype GA was associated with significant fall in eGFR levels in diabetic subjects compared with control ones.

Regarding ACE I/D polymorphism, the current study recorded that DD polymorphism was associated with increase in serum creatinine and cyst-c in diabetic patient group. According to Silveira et al.^18^, DD polymorphism was associated with DN and T2DM progression. This may be attributed to the functional role of DD polymorphism in the implication of pathological mechanisms through change of endothelial β cells, oxidative stress and renal microvascular complications. Also, Rahimi^28^ suggested that ethnicity represents one of the most important factors in determining ACE polymorphism role in DN susceptibility.

Heterozygous ID polymorphisms was associated with significant fall in eGFR levels in diabetic individuals compared with controls which is in accordance with Sauca et al.^29^ who revealed that in diabetic patients, ID polymorphism showed a slope in eGFR in T1DM and can be considered a determinant markers of DN progression. On the other hand, intermediate serum ACE activities were shown in heterozygous individuals (ID). That explained the association of higher ACE activity in patients with D allele with more extensive kidney damage^30^.

The current study found positive relation between duration of diabetes and level of serum cystatin-c in diabetic patient group (*p* < 0.027). That was in accordance with Saptoka et al.^6^ who found the same relation.

In addition, positive relation was found between urinary hemeoxygenase and serum glucose in diabetic patient group (*p* < 0.02). That may be due to oxidative effect of hyperglycemia in diabetic patients. This could be explained by tubular injury resulted from hyperglycemia as mentioned in Chang et al.^31^ who referred proximal tubule injury to high glucose transport state and mismatched local hypoxia in DM patients might be initiating factors to proximal tubular injury.

Lastly, in the present work, the crude AUC of urinary hemeoxygenase for prediction of the presence of DN was 0.356 suggesting that HO-1 in urine is urinary biomarker that can predict the risk for DN in T2DM diabetic patients.

While crude area under curve (AUC) of serum cystatin-c is 0.617, suggesting that serum cystatin-c could be blood biomarker that could predict early tubular injury in T2DM diabetic patients that is in line with Saptoka et al.^6^.

We can suggest the need of larger population for confirming the cut-off value of either blood cystatin-c or urinary hemeoxygenase for predicting DN unlike serum creatinine.

## Conclusion

Findings of this study indicated an association of serum cystatin-c with GG genotype of ACE G2350A in conjugation with DD polymorphism of ACE I/D gene as early predictor of tubular injury in T2DM diabetic. In addition, an increase in cystatin-c level was seen with individuals with longer history of diabetes. Therefore, assessment of serum cystatin-c seems to be a better diagnostic test for screening patient when serum creatinine level is inconclusive, especially, in certain individuals with longer duration of diabetes and other comorbidity.

## Subjects and methodology

### Subjects

This cross-sectional controlled study recruited 193 participants. They are grouped into 108 diabetic patients from out patient diabetes clinic in Kasr Al-Aini hospital and 85 controls. Control subjects were administrative and/or employee in different sectors in the National Research Centre in addition to other retired participants. The research protocol was approved by the Ethical Committee in the National Research Centre, Egypt with number (13132032021) in accordance with the Declaration of Helsinki. All the selected subjects signed an informed written consent for their participation in the study. A questionnaire including demographic, clinical and environmental history was filled out.

Seven ml of fasting blood sample was collected from each participant and divided into 2 tubes, an EDTA tube was used for determination of glycated hemoglobin (HbA_1_C) and ACE genes determination.

A second dry tube which was left at room temperature for 30 min, then centrifuged at 3000 rpm for 5 min to separate serum for the assessment of serum glucose, creatinine and cystatin-c. Also, a urine void sample was collected from each participants for urinary hemeoxygenase and creatinine levels measurement.

## Methods

### Biochemical measurements

#### Blood glucose

Blood glucose was estimated by the enzymatic colorimetric method according to Trinder^32^.

#### Glycated hemoglobin (HbA_1_c)

HbA_1_c (%) was performed by ion exchange method according to Bates^33^ according to the protocol supplied from MG, Science &Technology Center.

#### Determiantion of serum cystatin-c

Serum cystatin-c levels was measured using an enzyme-linked immunosorbent assay (ELISA) commercial kit (SinoGeneClon Biotech Co., Ltd).

#### Measurement of urinary hemeoxygenase

Urinary hemeoxygenase levels was measured using an enzyme-linked immunosorbent assay (ELISA) commercial kit (SinoGeneClon Biotech Co., Ltd).

#### Measurement of serum and urinary creatinine

Determination of serum and urinary creatinine (Cr) were carried out by using the Jaffe kinetic method without deproteinization according to Bartels^34^. Urine creatinine was estimated to adjust the values of the urinary hemeoxygenase.

#### Calculation of estimated glomerular filtration rate (eGFR) according to Inker et al.^35^

Estimated GFR was calculated using Chronic Kidney Disease-Epidemiology Collaboration (CKD-EPI) equation; GFR (mL/min/1.73 m^2^) = 141 × min (S_Cr/K_, 1)^α^ × max (S_Cr/K_, 1)^−1.209^ × 0.993^Age^ × 1.018^(if female)^ × 1.159^(if black)^ (where, K = 0.7 [if female] or 0.9 [if male] and α = 0.329 [if female] or 0.411 [if male]).

#### Genetic determination of ACE I/D and ACE G2350A genotypes

DNA was extracted from peripheral blood mononuclear cells using using a QIAamp® DNA Blood Mini kit (Qiagen, Hilden, Germany), according to the manufacturer's protocol for genetic assay:

- ACE gene (I/D) polymorphism was performed by PCR methods according to Rigat et al.^36^. Ten pmoles of each primer: sense oligo 5′ CTGGAGACCACTCCCATCC1TTCT 3′ and anti-sense oligo: 5′ GATGTGGCCATCACATTCGTCAGAT3′ was set in reaction of 50 µL final volume. This volume contains 1U of Taq polymerase, 0.5 mM of each dNTP (Promega), 3 mM MgCl2, 50 mM KCI, 10 mM Tris-HCI pH 8.4. Amplification program (using a PTC-100 thermal cycler) was 30 cycles (denaturation at 94 °C for 1 min, annealing at 58 °C for 1 min, and extension at 72 °C for 2 min. Identification of PCR product was visualized after agarose gel electrophoresis and ethidium bromide staining. A fragment of 190 bp was shown in the absence of the insertion (D) while in the presence of the insertion (I), a fragment of 490 bp was observed. A heterozygote, third fragment, with an intermediate molecular weight showing both fragment at 190 bp and 490 bp corresponds to ID genotype. The patients were then grouped into the DD, II and ID genotypes respectively.
- (ACE) gene G2350A was assayed by PCR-RFLP according to Mahmood et al.^37^. The PCR primers sequences were as follows:
  Forward primer: 5′-CTGACGAATGTGATGGCCGC-3′ and Reverse primer 5′-TTGATGAGTTCCACGTATTTCG-3′. The PCR conditions was carried out: initial denaturation at 95 °C for 5 min followed by 35 cycles where 30 s for denaturation at 94 °C, 30 s at 58 °C for annealing, and 30 s at 72 °C for extension, and 10 min at 72 °C a for final extension time. The PCR products were digested with 5 U of fast digest BstU1 at 60 °C for 2 h. Digested fragments were separated by electrophoresis on 3.5% agarose gel and identified by ethidium bromide staining using ultraviolet trans-illumination. Allele G2350 was visualized as a 122-bp fragment and allele A2350 as 100-bp and 22-bp fragments and GA genotype appear as 122–100 and 22 bp.

### Statistical analysis

Analysis of data was performed through SPSS program version 18. Expression of data was as mean ± SD. Differences between groups was compared by using Independent student’s *t*-test. Significance was set at *p* < 0.05. Relation between variables was studied by Pearson's correlation. The χ^2^ test was used to examine the differences in genotype distribution between patients and controls groups. Receiver Operating Characteristics (ROC) analysis was done for calculating the Area Under the Curve (AUC) for serum cystatin-c and urinary HO-1 to find the best cut-off values to identify diabetic Nephropathy.

### Ethical approval

The study was conducted in accordance with the Declaration of Helsinki, and ethical approval was obtained from National Research Centre Ethical committee (NRCEC-13132032021) of National Research Centre, Egypt.

### Informed consent

Informed consent was signed from all participants included in the study.

## Data Availability

Data are not publicly available due to privacy reasons and data supporting the fndings of this study are available from the corresponding author upon request.
